# Curcumin: A Review of Its’ Effects on Human Health

**DOI:** 10.3390/foods6100092

**Published:** 2017-10-22

**Authors:** Susan J. Hewlings, Douglas S. Kalman

**Affiliations:** 1 Department of Nutrition, Central Michigan University, Mount Pleasant, MI 48859, USA; 2 Substantiation Sciences, Weston, FL 33332, USA; 3 Health and Human Performance, Nova Southeastern University, Fort Lauderdale, FL 33314, USA; 4 Nutrition Research Division, QPS, Miami, FL 33143, USA; dougkalman@gmail.com

**Keywords:** curcumin, turmeric, antioxidant, anti-inflammatory, polyphenol

## Abstract

Turmeric, a spice that has long been recognized for its medicinal properties, has received interest from both the medical/scientific world and from culinary enthusiasts, as it is the major source of the polyphenol curcumin. It aids in the management of oxidative and inflammatory conditions, metabolic syndrome, arthritis, anxiety, and hyperlipidemia. It may also help in the management of exercise-induced inflammation and muscle soreness, thus enhancing recovery and performance in active people. In addition, a relatively low dose of the complex can provide health benefits for people that do not have diagnosed health conditions. Most of these benefits can be attributed to its antioxidant and anti-inflammatory effects. Ingesting curcumin by itself does not lead to the associated health benefits due to its poor bioavailability, which appears to be primarily due to poor absorption, rapid metabolism, and rapid elimination. There are several components that can increase bioavailability. For example, piperine is the major active component of black pepper and, when combined in a complex with curcumin, has been shown to increase bioavailability by 2000%. Curcumin combined with enhancing agents provides multiple health benefits. The purpose of this review is to provide a brief overview of the plethora of research regarding the health benefits of curcumin.

## 1. Introduction

Turmeric is a spice that has received much interest from both the medical/scientific worlds as well as from the culinary world. Turmeric is a rhizomatous herbaceous perennial plant (*Curcuma longa*) of the ginger family [1]. The medicinal properties of turmeric, the source of curcumin, have been known for thousands of years; however, the ability to determine the exact mechanism(s) of action and to determine the bioactive components have only recently been investigated [2]. Curcumin (1,7-bis(4-hydroxy-3-methoxyphenyl)-1,6-heptadiene-3,5-dione), also called diferuloylmethane, is the main natural polyphenol found in the rhizome of *Curcuma longa* (turmeric) and in others *Curcuma* spp. [3]. *Curcuma longa* has been traditionally used in Asian countries as a medical herb due to its antioxidant, anti-inflammatory [4], antimutagenic, antimicrobial [5,6], and anticancer properties [7,8].

Curcumin, a polyphenol, has been shown to target multiple signaling molecules while also demonstrating activity at the cellular level, which has helped to support its multiple health benefits [2]. It has been shown to benefit inflammatory conditions [9], metabolic syndrome [10], pain [11], and to help in the management of inflammatory and degenerative eye conditions [12,13]. In addition, it has been shown to benefit the kidneys [14]. While there appear to be countless therapeutic benefits to curcumin supplementation, most of these benefits are due to its antioxidant and anti-inflammatory effects [2,9]. Despite its reported benefits via inflammatory and antioxidant mechanisms, one of the major problems with ingesting curcumin by itself is its poor bioavailability [15], which appears to be primarily due to poor absorption, rapid metabolism, and rapid elimination. Several agents have been tested to improve curcumin’s bioavailability by addressing these various mechanisms. Most of them have been developed to block the metabolic pathway of curcumin in order to increase its bioavailability. For example, piperine, a known bioavailability enhancer, is the major active component of black pepper [16] and is associated with an increase of 2000% in the bioavailability of curcumin [17]. Therefore, the issue of poor bioavailability appears to be resolved by adding agents such as piperine that enhance bioavailability, thus creating a curcumin complex.

Curcumin is being recognized and used worldwide in many different forms for multiple potential health benefits. For example, in India, turmeric—containing curcumin—has been used in curries; in Japan, it is served in tea; in Thailand, it is used in cosmetics; in China, it is used as a colorant; in Korea, it is served in drinks; in Malaysia, it is used as an antiseptic; in Pakistan, it is used as an anti-inflammatory agent; and in the United States, it is used in mustard sauce, cheese, butter, and chips, as a preservative and a coloring agent, in addition to capsules and powder forms. Curcumin is available in several forms including capsules, tablets, ointments, energy drinks, soaps, and cosmetics [2]. Curcuminoids have been approved by the US Food and Drug Administration (FDA) as “Generally Recognized As Safe” (GRAS) [2], and good tolerability and safety profiles have been shown by clinical trials, even at doses between 4000 and 8000 mg/day [18] and of doses up to 12,000 mg/day of 95% concentration of three curcuminoids: curcumin, bisdemethoxycurcumin, and demethoxycurcumin [19].

It is the purpose of this review to provide a brief overview of the plethora of research regarding the potential health benefits of curcumin. Due to the extent of the literature, we have chosen to focus on the benefits associated with some common health conditions and on benefits in healthy people rather than to review the extensive literature related to cancer and other disease states. For a comprehensive review of curcumin’s effects on cancer, please see the paper by Kunnumakkara et al. 2017 [20].

## 2. Mechanisms of Action

### 2.1. Antioxidant

Antioxidant and anti-inflammatory properties are the two primary mechanisms that explain the majority of the effects of curcumin on the various conditions discussed in this review [21,22]. Curcumin has been shown to improve systemic markers of oxidative stress [23]. There is evidence that it can increase serum activities of antioxidants such as superoxide dismutase (SOD) [24,25,26]. A recent systematic review and meta-analysis of randomized control data related to the efficacy of supplementation with purified curcuminoids on oxidative stress parameters—indicated a significant effect of curcuminoids supplementation on all investigated parameters of oxidative stress including plasma activities of SOD and catalase, as well as serum concentrations of glutathione peroxidase (GSH) and lipid peroxides [23]. It is noteworthy to point out that all of the studies included in the meta-analysis utilized some sort of formulation to overcome bioavailability challenges, and four out of the six used piperine. Curcumin’s effect on free radicals is carried out by several different mechanisms. It can scavenge different forms of free radicals, such as reactive oxygen and nitrogen species (ROS and RNS, respectively) [25]; it can modulate the activity of GSH, catalase, and SOD enzymes active in the neutralization of free radicals [21,22]; also, it can inhibit ROS-generating enzymes such as lipoxygenase/cyclooxygenase and xanthine hydrogenase/oxidase [21]. In addition, curcumin is a lipophilic compound, which makes it an efficient scavenger of peroxyl radicals, therefore, like vitamin E, curcumin is also considered as a chain-breaking antioxidant [27].

### 2.2. Anti-Inflammatory

Oxidative stress has been implicated in many chronic diseases, and its pathological processes are closely related to those of inflammation, in that one can be easily induced by another. In fact, it is known that inflammatory cells liberate a number of reactive species at the site of inflammation leading to oxidative stress, which demonstrates the relationship between oxidative stress and inflammation [28]. In addition, a number of reactive oxygen/nitrogen species can initiate an intracellular signaling cascade that enhances pro-inflammatory gene expression. Inflammation has been identified in the development of many chronic diseases and conditions [10,19,29,30]. These diseases include Alzheimer’s disease (AD), Parkinson’s disease, multiple sclerosis, epilepsy, cerebral injury, cardiovascular disease, metabolic syndrome, cancer, allergy, asthma, bronchitis, colitis, arthritis, renal ischemia, psoriasis, diabetes, obesity, depression, fatigue, and acquired immune deficiency syndromeAIDS [10]. Tumor necrosis factor α (TNF-α) is a major mediator of inflammation in most diseases, and this effect is regulated by the activation of a transcription factor, nuclear factor (NF)-κB. Whereas TNF-α is said to be the most potent NF-κB activator, the expression of TNF-α is also regulated by NF-κB. In addition to TNF-α, NF-κB is also activated by most inflammatory cytokines; gram-negative bacteria; various disease-causing viruses; environmental pollutants; chemical, physical, mechanical, and psychological stress; high glucose; fatty acids; ultraviolet radiation; cigarette smoke; and other disease-causing factors. Therefore, agents that downregulate NF-κB and NF-κB–regulated gene products have potential efficacy against several of these diseases. Curcumin has been shown to block NF-κB activation increased by several different inflammatory stimuli [10]. Curcumin has also been shown to suppress inflammation through many different mechanisms beyond the scope of this review, thereby supporting its mechanism of action as a potential anti-inflammatory agent [10].

## 3. Arthritis

One such disease associated with inflammation, both chronic and acute, is osteoarthritis (OA), a chronic joint condition. It affects over 250 million people worldwide, leading to increased healthcare costs, impairment in activities of daily living (ADL), and ultimately decreased quality of life [31,32]. Although OA was once considered primarily a degenerative and non-inflammatory condition, it is now recognized as having inflammatory aspects, including elevated cytokine levels, as well as potentially being connected with systemic inflammation [33,34]. While there is no cure, there are several pharmaceutical options for treatment; however, many are costly and have undesirable side effects. Therefore, there is increased interest in alternative treatments including dietary supplements and herbal remedies [35]. Several studies have shown the anti-arthritic effects of curcumin in humans with OA and rheumatoid arthritis (RA) [36,37,38,39]. In a randomized double-blind placebo-controlled trial, 40 subjects with mild-to-moderate degree knee OA were randomly assigned to receive either curcuminoid (500 mg/day in three divided doses; *n* = 19) with 5 mg piperine added to each 500-mg dose or a matched placebo (*n* = 21) for six weeks. There were significantly greater reductions in the visual analog scale (VAS) (*p* < 0.001), Western Ontario and McMaster Universities Osteoarthritis Index (WOMAC) scores (*p* = 0.001), and Lequesne’s pain functional index (LPFI) (*p* = 0.013) scores in the treatment group compared with the placebo group. When comparing the WOMAC subscales, there were significant improvements in the pain and physical function scores (*p* < 0.001), but not in the stiffness score [40]. There was also a decrease in systemic oxidative stress, as measured via serum activities of SOD and concentrations of reduced GSH and malonedialdehyde (MDA), in subjects receiving the treatment as compared to the placebo [11]. These improvements were not associated with changes in circulating cytokines. The authors suggest that the lack of changes in circulating cytokines, despite improvements in pain, may be because in OA, inflammatory markers in the synovial fluid may be more likely elevated than systemic markers, whereas in RA, systemic markers may be more likely to be increased. Therefore, they suggest that is more plausible that the beneficial effects of curcuminoids in OA are because of local anti-inflammatory effects rather than systemic effects. In addition, the time period of supplementation may not have been long enough. In a longer (eight months) randomized control trial, 50 subjects diagnosed with OA were assigned to receive either standard treatment as prescribed by their physician or standard treatment plus two 500-mg tablets daily consisting of a natural curcuminoid mixture (20%), containing phosphatidyl-choline (40%) and microcrystalline cellulose (40%). WOMAC, physical function, and stiffness scores decreased significantly (*p* < 0.05) in the treatment group compared to the control. In addition, the treatment group showed significant decreases in all markers of inflammation (soluble CD40 ligand(sCD40L), interleukin 1 beta (IL-1β), interleukin 6 (IL-6), soluble vascular cell adhesion molecule 1 (sVCAM-1), and erythrocyte sedimentation rate (ESR) comparing baseline to follow-up, while the control group did not [37]. This study had both groups maintaining standard care, which does not address the question of whether or not supplementation with curcumin can be used instead of standard management such as nonsteroidal anti-inflammatory drugs (NSAIDS). To address this question, 367 primary knee osteoarthritis patients with a pain score of 5 or higher were randomized to receive ibuprofen 1200 mg/day or *C. domestica* extracts 1500 mg/day for four weeks. The mean of all WOMAC scores at weeks 0, 2, and 4 showed significant improvement when compared with the baseline in both groups. After using the noninferiority test, the mean difference (95% confidence interval) of WOMAC total, WOMAC pain, and WOMAC function scores at week 4 adjusted by values at week 0 of *C. domestica* extracts were non-inferior to those for the ibuprofen group (*p* = 0.010, *p* = 0.018, and *p* = 0.010, respectively), indicating that those taking the curcumin and those taking the ibuprofen experienced the same benefits. The group taking the NSAIDS did experience more gastrointestinal issues. This suggests that curcumin may offer an alternative to NSAIDS for patients with OA seeking treatment but experiencing negative side effects [12]. This was supported by results from a pilot study showing that a dose of 2 g of curcumin had an analgesic effect in subjects with acute pain but without a diagnosis of OA. At this dose, the activity was higher than that associated with 500 mg of acetaminophen, while a lower dose (1.5 g, 300 mg of curcumin) gave only transient and often inadequate relief of pain, indicative of suboptimal therapeutic plasma concentrations. The analgesic effect of the dose achieved significance only 2 h after administration, similar to that observed for acetaminophen. In contrast, the NSAID was more rapidly acting, with the strongest pain relief being reported one hour after administration but with significant gastrointestinalsymptoms. This supports the use of 2 g (higher than needed for inflammation) curcumin for relief of pain as a potential alternative to NSAIDS [41].

Regardless of the mechanism by which curcumin elicits its effects, it does appear to be beneficial to several aspects of OA, as suggested by a recent systematic review and meta-analysis that concluded: “This systematic review and meta-analysis provided scientific evidence that 8–12 weeks of standardized turmeric extracts (typically 1000 mg/day of curcumin) treatment can reduce arthritis symptoms (mainly pain and inflammation-related symptoms) and result in similar improvements in the symptoms as ibuprofen and diclofenac sodium. Therefore, turmeric extracts and curcumin can be recommended for alleviating the symptoms of arthritis, especially osteoarthritis” [42].

## 4. Metabolic Syndrome

The idea that curcumin can attenuate systemic inflammation has implications beyond arthritis, as systemic inflammation has been associated with many conditions affecting many systems. One such condition is Metabolic syndrome (MetS), which includes insulin resistance, hyperglycemia, hypertension, low high-density lipoprotein cholesterol (HDL-C), elevated low-density lipoprotein cholesterol (LDL-C), elevated triglyceride levels, and obesity, especially visceral obesity. Curcumin has been shown to attenuate several aspects of MetS by improving insulin sensitivity [43,44], suppressing adipogenesis [45], and reducing elevated blood pressure [46], inflammation [47], and oxidative stress [48,49]. In addition, there is evidence that curcuminoids modulate the expression of genes and the activity of enzymes involved in lipoprotein metabolism that lead to a reduction in plasma triglycerides and cholesterol [50,51,52] and elevate HDL-C concentrations [53]. Both overweight and obesity are linked to chronic low-grade inflammation; although the exact mechanisms are not clear, it is known that pro-inflammatory cytokines are released. These cytokines are thought to be at the core of the complications associated with diabetes and cardiovascular disease. Therefore, addressing inflammation is important. In a randomized double-blind placebo-controlled trial with a parallel-group design, 117 subjects with MetS received either 1 g curcumin plus 10 mg piperine to increase absorption or a placebo plus 10 mg piperine for eight weeks. Within-group analysis revealed significant reductions in serum concentrations of TNF-α, IL-6, transforming growth factor beta (TGF-b), and monocyte chemoattractant protein-1 ( MCP-1) following curcumin supplementation (*p* < 0.001). In the placebo group, serum levels of TGF-b were decreased (*p* = 0.003) but those of IL-6 (*p* = 0.735), TNF-α (*p* = 0.138), and MCP-1 (*p* = 0.832) were not. Between-group comparison suggested significantly greater reductions in serum concentrations of TNF-α, IL-6, TGF-b, and MCP-1 in the curcumin versus the placebo group (*p* < 0.001). Apart from IL-6, changes in other parameters remained statistically significant after adjustment for potential confounders, including changes in serum lipids and glucose levels, as well as the baseline serum concentration of the cytokines. The results of this study suggest that curcumin supplementation significantly decreases serum concentrations of pro-inflammatory cytokines in subjects with MetS [11]. In addition, the study looked at the cholesterol-lowering properties and found that curcuminoids were more effective than the placebo in reducing serum LDL-C, non-HDL-C, total cholesterol, triglycerides, and lipoprotein a (Lp(a)), in addition to elevating HDL-C concentrations. However, changes in serum LDL-C levels were found to be comparable between the study groups. The effects of curcuminoids on triglycerides, non-HDL-C, total cholesterol, and Lp(a) remained significant after adjustment for baseline values of lipids and body mass index [54]. From the same study, the authors also reported markers of oxidative stress. There was a significant improvement in serum SOD activities (*p* < 0.001) and reduced MDA (*p* < 0.001) and C-reactive protein (CRP) (*p* < 0.001) concentrations in the group receiving the curcumin with piperine compared to the placebo group. Their secondary purpose was to perform a meta-analysis of data from all randomized controlled trials in order to estimate the effect size of curcuminoids on plasma CRP concentrations. Quantitative data synthesis revealed a significant effect of curcuminoids vs. placebo in reducing circulating CRP concentrations The authors concluded that short-term supplementation with a curcuminoid-piperine combination significantly improves oxidative and inflammatory status in patients with MetS. Curcuminoids could therefore be regarded as natural, safe, and effective CRP-lowering agents [55].

Inflammatory cytokines were also measured in the above study. Mean serum IL-1β (*p* = 0.042), IL-4 (*p* = 0.008), and vascular endothelial growth factor (VEGF) (*p* = 0.01) were found to be significantly reduced by curcumin therapy. In contrast, no significant difference was observed in the concentrations of IL-2, IL-6, IL-8, IL-10, interferon gamma(IFNγ), epidermal growth factor (EGF), and MCP-1. The authors suggest that the findings indicate that curcumin may exert immunomodulatory effects via altering the circulating concentrations of IL-1β, IL-4, and VEGF [56].

In a randomized double-blind placebo-controlled crossover trial, 36 obese adults received either 1 g curcumin and 10 mg piperine or a placebo for 30 days followed by a two-week washout period, after which they received the other treatment. A significant reduction in serum triglyceride concentrations was observed, but the treatment did not have a significant influence on serum total cholesterol, LDL-C, HDL-C, and high-sensitivity C-reactive protein (hs-CRP) concentrations, nor on body mass index (BMI) and body fat. The authors suggest that the short supplemental period, lack of control of diet, and the low supplemental dose may explain why these results conflict previous reports [50].

## 5. Healthy People

To date, the majority of curcumin studies in humans have been in populations with existing health problems. Perhaps this is because studies on healthy people can be challenging in that benefits may not be as immediate and measurable if biomarkers are normal at baseline. Therefore, following subjects over time may provide the best insight into any potential health benefits in healthy people, although such studies can be time-consuming and costly. Making cross-comparisons between the few studies that have been done can be difficult because studies have used varying doses, often as high as 1 g [57,58]. It should be noted that this would be considered a high dose only because it is higher than what most people could obtain from consuming the spice itself [49]. One study on healthy adults aged 40–60 years used an 80 mg/day dose of a lipidated form of curcumin. Subjects were given either curcumin (*N* = 19) or a placebo (*N* = 19) for four weeks. The treatment was 400 mg powder per day containing 80 mg curcumin. Blood and saliva were taken before and after the four weeks. Curcumin significantly lowered triglyceride levels but not total cholesterol, LDL, or HDL levels. There was a significant increase in nitrous oxide (NO) and in soluble intercellular adhesion molecule 1 (sICAM), a molecule linked to atherosclerosis. Inflammation-related neutrophil function increased, as measured by myeloperoxidase concentration, but c-reactive protein and ceruloplasmin did not. There was a decrease in salivary amylase activity, which can be a marker of stress, and an increase in salivary radical scavenging capacities and plasma antioxidant enzyme catalase, but not in super oxide dismutase or glutathione peroxidase. In addition, there was a decrease in beta amyloid plaque, a marker of brain aging, and in plasma alanine amino transferase activities, a marker of liver injury. This indicates that a relatively low dose of curcumin can provide health benefits for people that do not have diagnosed health conditions [51].

In a randomized double-blind placebo-controlled trial, the acute (1 and 3 h after a single dose), chronic (four weeks), and acute-on-chronic (1 and 3 h after single dose following chronic treatment) effects of solid lipid curcumin formulation on cognitive function, mood, and blood biomarkers in 60 healthy adults aged 60–85 years were examined. The curcumin formulation was 400 mg, approximately 80 mg curcumin in a solid lipid formulation with the remaining weight comprised of commonly used pharmaceutical excipients and small amounts of other curcuminoids present in turmeric extract. One hour after administration, curcumin significantly improved performance on sustained attention and working memory tasks, compared with the placebo. Working memory and mood (general fatigue and change in state calmness, contentedness, and fatigue induced by psychological stress) were significantly better following chronic treatment. A significant acute-on-chronic treatment effect on alertness and contentedness was also observed. Curcumin was associated with significantly reduced total and LDL cholesterol [59].

Another study examined whether supplementation with curcumin and Boswellia serrata (BSE) gum resin for three months could affect plasma levels of markers of oxidative stress, inflammation, and glycation in 47 male healthy master cyclists. All subjects were instructed to follow a Mediterranean diet with 22 subjects receiving a placebo and 25 receiving 50 mg of turmeric, corresponding to 10 mg of curcumin, as well as 140 mg of Boswellia extract, corresponding to 105 mg of Boswellia acid for 12 weeks. There was a positive effect observed on glycoxidation and lipid peroxidation in healthy male master athletes [60]. This study indicates the potential for combining curcumin with other agents to achieve health benefits.

Perhaps another challenge to interpreting studies on healthy people is determining the definition of healthy, especially when considering that people who do not have an official diagnosis may still participate in activities or experience situations whereby they challenge their daily physiological homeostasis. For example, an exercise routine that one is not used to can cause inflammation, oxidative challenges, and resulting soreness. In a recent study, 28 healthy subjects that did not participate in resistance training were randomly assigned to receive either curcumin (400 mg/day) for two days before and four days after participating in an eccentric exercise designed to induce muscle soreness. Curcumin supplementation resulted in significantly smaller increases in creatine kinase (CK) (−48%), TNF-α (−25%), and IL-8 (−21%) following exercise compared to the placebo. No significant differences in IL-6, IL-10, or quadriceps muscle soreness between conditions were observed. The findings demonstrated that the consumption of curcumin reduced biological inflammation, but not subjective quadriceps muscle soreness during recovery from exercise. This may help to decrease recovery time, thus improving performance during subsequent exercise sessions [61].

In a similar randomized placebo-controlled single-blind pilot trial, 20 male healthy, moderately active volunteers were randomized to receive either 1 g curcumin twice daily (200 mg curcumin twice a day ) or a placebo 48 h prior to and 24 h after a downhill running test. Subjects in the curcumin group reported significantly less pain in the right and left anterior thigh. Significantly fewer subjects in the curcumin group had MRI evidence of muscle injury in the posterior or medial compartment of both thighs. Increases in markers of muscle damage and inflammation tended to be lower in the curcumin group, but significant differences were only observed for interleukin-8 at 2 h after exercise. No differences in markers of oxidative stress and muscle histology were observed. These results further support that curcumin may be beneficial to attenuate exercise-induced muscle soreness (DOMS) [62].

A study by Delecroix et al. offers further support. They reported that 2 g of curcumin and 20 g of piperine supplementation can help offset some of the physiological markers of muscle soreness after an intense workout in elite rugby players [63].

In addition to acute physical stresses, humans may also suffer from periods of anxiety or depression which are sub clinical, but may still benefit from treatments that can decrease the symptoms. In a randomized double blind cross-over trial, 30 obese adults received curcuminoids (1 g/day) or a placebo for 30 days, and then after a two-week washout period, crossed over to the alternate regimen. The curcumin was a 500-mg C3 Complex^®^ (standardized powder extract obtained from Alleppey finger turmeric containing a minimum 95% concentration of three curcuminoids: curcumin, bisdemethoxycurcumin, and demethoxycurcumin) plus 5 mg bioperine^®^ per serving to enhance absorption. Beck Anxiety Inventory (BAI) and Beck Depression Inventory (BDI) scales were filled out for each participant at baseline and after four, six, and 10 weeks of supplementation. Mean BAI score was found to be significantly reduced following curcumin therapy (*p* = 0.03). However, curcumin supplementation did not exert any significant impact on BDI scores. This study suggests that curcumin has a potential anti-anxiety effect in otherwise healthy obese people [64].

## 6. Side Effects

Curcumin has a long established safety record. For example, according to JECFA (The Joint United Nations and World Health Organization Expert Committee on Food Additives) and EFSA (European Food Safety Authority) reports, the Allowable Daily Intake (ADI) value of curcumin is 0–3 mg/kg body weight [65]. Several trials on healthy subjects have supported the safety and efficacy of curcumin. Despite this well-established safety, some negative side effects have been reported. Seven subjects receiving 500–12,000 mg in a dose response study and followed for 72 h experienced diarrhea, headache, rash, and yellow stool [19]. In another study, some subjects receiving 0.45 to 3.6 g/day curcumin for one to four months reported nausea and diarrhea and an increase in serum alkaline phosphatase and lactate dehydrogenase contents [66].

## 7. Conclusions

Curcumin has received worldwide attention for its multiple health benefits, which appear to act primarily through its anti-oxidant and anti-inflammatory mechanisms. These benefits are best achieved when curcumin is combined with agents such as piperine, which increase its bioavailability significantly. Research suggests that curcumin can help in the management of oxidative and inflammatory conditions, metabolic syndrome, arthritis, anxiety, and hyperlipidemia. It may also help in the management of exercise-induced inflammation and muscle soreness, thus enhancing recovery and subsequent performance in active people. In addition, a relatively low dose can provide health benefits for people that do not have diagnosed health conditions.
